# A Case of Monstrosity

**Published:** 1867-03

**Authors:** L. I. Marvin

**Affiliations:** Northampton, New York


*A Case of Monstrosity.* By L. I. Marvin, M. D., of Northampton, New York.

In the month of July, 1859, I was called in haste to visit a woman who was reported to have been in labor two days and two nights, and was said to be at least in a very critical condition. When I arrived, the family physician was present.

The patient was an Irish woman, 28 years of age, and it was her first accouchment. She was a short, thick, and what might be called, in common phrase, a chubby woman. The attending physician informed me that he had been with her forty-eight hours, and her pains had been regular, pressing, and severe, and yet no advancement in her labor had been apparent.

On examination, I found the head presenting naturally, and down within the pelvis. The external parts were preternaturally dilated, so that I could easily introduce both hands each side of the head to the neck of the child, thereby forming a sufficient hold for all the traction necessary during her pains, which were frequent and very severe. I assisted her for twenty or thirty minutes, with all the traction I dared to apply, but without any advancement in the labor.

Her strength was failing rapidly, and no advancement of the head, but the same firm and solid resistance to advancement continuing, I perceived at once that delivery must be effected soon, in order to save the patient.

I commenced rotating the head within the pelvis until I twisted it off and brought it away.

On further examination, an arm presented, and by the same traction I had previously used, the same firm and unyielding resistance remained, though her throes were strong and frequent. I then removed the arm by twisting it off at the shoulder.joint without difficulty.

I then introduced my hand and brought down a leg, which being secured by an assistant, I succeeded in bringing down another, together with the pelvis. I then supposed I was, as I remarked to the attending physician, *out of the woods*, and would soon be through. I was disappointed; for after a powerful throe I found that the same permanent resistance continued.

In the absence of a pain, I introduced my hand, and seized another leg and brought it down and secured it as before. I then brought down another leg with another pelvis.

Thus I had four legs and two pelvises in the world. My patient much exhausted, I administered cordials and stimulants, and when sufficiently restored, I succeeded in delivering her.

I placed the head and arm in their proper places on a table-cloth, with the rest of the parts, and it presented one of the most interesting specimens of monstrosity I ever saw, or have read of; and I doubt if another case of the kind is on record. Two female children, or fœtuses, fully grown, perfectly formed, and fully developed in every limb and external part. Two heads, four arms, a double thorax, one common abdomen, two pelvises. External organs all perfectly and fully developed, and equal in both. It weighed with the table cloth in which it was bound up, eighteen and three-quarter pounds.

I have seen many cases of monstrosity of children, or fœtuses, but none that approached the regularity and full development of this in every external organ. Heads, faces, and features were perfectly natural, and appeared as if two full-grown fœtuses had lain crosswise, and to use a mechanical phrase, had been welded together—the two abdomens in one.

I offered large inducements to the parents, to allow me to preserve this most remarkable specimen of monstrosity; but as they were Catholics, no overtures were of any avail, as it would in their view be sacrilege, and it must be buried in consecrated ground. My principal object in obtaining the specimen was to ascertain whether there were two sets of abdominal viscera or only one. Professor March, of the Albany Medical College, was here a short time after, and I related the case to him. He was very desirous of procuring the specimen, but it was in the heat of summer, and probably decomposition had taken place, and it was abandened. The woman recovered, and has had four children since.—*Med. and Surg. Reporter.*

